# The Teeth of Different People

**Published:** 1885-08

**Authors:** Parsons Shaw

**Affiliations:** Manchester, England.


ARTICLE II.

### THE TEETH OF DIFFERENT PEOPLE.

BY PARSONS SHAW, D. D. S., MANCHESTER, ENGLAND.

In disregard of the great law that we should "not judge by appearances but judge righteously" (which is a fundamental one in all scientific investigations, ) perhaps there is no subject on which there is more dogmatic assertion, with less knowledge of the facts, than on the conditions and comparative value of the teeth. And these assertions have become incorporated into our text books, and pass current,when they are in most cases merely the result of ignorance, prejudice, and misrepresentations for a settled purpose. As I have had opportunities for observing the teeth of Americans in different parts of the Union, and the teeth of people from different parts of Europe, as well as some of the wooly-haired Africans, I will record my observations and conclusions, and hope that others will follow with their experience until this matter is settled on a solid basis.

It is a favorite way in Europe of accounting for the superiority of American dentistry by assuming that dentists are more needed in America than elsewhere, owing to the more rapid decay of the teeth. When we get to understand the meaning of this assumption, we find it to be only a part and parcel of a great system. It is taking for granted by European authorities that everything must be wrong in America, as the government, fiscal policy, social life, religion and morals are, according to their views, all based on a false foundation. And then it is argued that, as under pernicious institutions no people can prosper, it is, therefore, natural to find a gradual decay of the Americans, politically, morally and physically. The few who do not quite condemn American institutions attribute this assumed deterioration to the climate; but the degeneration of the Americans in general, and of the descendants of the Puritans in particular, is almost universally taken as a settled fact. The reason why it is not more apparent is owing, so we are assured, to the new blood brought in by emigration. When the average American comes in contact with these views he has no suspicion of their real meaning, and is quite too apt to adopt them without reflection. Or if he begins to make investigations it is usually among his foreign patients, by whom he is misled either through their preconceived notions of American degeneracy, or their conceit. We know that teeth decay a great deal before patients are aware of it, and it is a common thing for them to say their teeth have gone within a few months, when the slightest investigation shows they have been decaying, more or less, for years. It is a common experience in my practice for a foreigner to assure me his teeth never decayed until he came to England, simply because it was after he came here that he happened to have his first toothache, and never had been to a dentist to ascertain the real condition of his teeth. For the same reason the Englishman is certain his teeth never decayed until he went to live somewhere out of England; and the man from the South is equally certain his teeth were all sound when he came to the North; and the man from the North avows he never had a speck of decay on his teeth until he went into the South; and so it goes all round the compass. It is, therefore, no evidence when the foreigner tells the Americans his teeth were all sound when he came to America, and that teeth do not decay in the "old country" as they do there. His assurances are based upon ignorance of the progress of decay in his teeth, and of the condition they would have been in if he had never emigrated, strengthened by the preconceived notions which grew out of his patriotic bounce. In so readily accepting these errors I am not certain there is not a good deal of something of the same sort of unconscious patriotism in the American who takes for granted the foreigner's view of American teeth. I suspect the logic is something like this. Its postulate is the common and vulgar notion that a higher civilization is only obtained by a corresponding loss of physical powers. Therefore, if we assume that the American has a higher civilization than that represented by the foreigner who comes to his country, it follows on his postulate that his teeth decay sooner.

Just as it is a mistake that higher civilization implies physical degeneracy, so it is an error to assume that the classes who hereditarily live by labor in old and settled countries are physically superior to the hereditarily cultured classes. It is only in new communities that all sorts of people are mixed up together, and wherever the people are settled down in to the regular routine of life they are eventually divided into classes (not by the possession of wealth, patents of nobility that are real or assumed, or by any other artificial means, but) by a course of natural selection based upon immutable laws. There are instincts, modes of thought, motives, ideas of what promotes happiness, and dietary and sanitary regulations which purify and elevate; and there are those which not only prevent any elevation, but must degrade. So that in the same community we find people with entirely different modes of thought, incentives to action, and consequent results. It is inevitable, therefore, that in the long course of time different classes should arise with fixed types, which are intensified by the constant intermarriage of those of the same blood, social standing and character. No American who has not come in contact with the varions classes in Europe, and not had an opportunity to study their characteristics, can have any idea of the radical difference between them, owing to the wide difference in the prevailing notions which govern all their actions. There is but little community of sentiment, except in their common humanity. This is almost at once revealed to those who have to treat their diseases. The superior classes are invariably grateful, patient, and strictly obedient to all commands as to diet, sanitary arrangements and medicines, while the lower orders seem to hate obedience, and systematically disregard the most imperative instructions, especially as to diet, and preclude the possibility of the exercise of any feeling of gratitude by the almost invariable habit of endeavoring to make it appear, in every transaction of life, that it is they who are conferring the favor. The inevitable result of the natural selection I have named is that those who obey the mental, moral and physical laws of life arise to the top, while those who habitually disregard either set of these laws sink to the bottom of the social scale. It is true there are at work, at all times, unexpected and unpreventable circumstances which appear to set aside this natural selection, if not altogether to defy it. And the struggles of such of the lower orders as have got elevated out of their real sphere by some stroke of luck, to maintain themselves in their unnatural position by the innumerable devices to which they resort, are apt to lead their less fortunate and unreflective fellows of the same order to imagine that men are lifted up by means of these low devices and mere assumption. But they are only a part of the system which eventually still more degrades; and it remains none the less certain that when a class rises to the top of the social scale and remains permanently there it is because they obey, on the whole the great moral and physical laws; and that the lower classes remain such because they are wedded to opinions, appetites, instincts, prejudices modes of thought and ways of life which cannot elevate, but must degrade. In accordance with the foregoing, I have found that those who belong by inheritance, to the upper classes, all over Europe, are in almost every way the superiors, mentally, morally and physically, of the permanently lower orders. The English gentleman has always beaten the common fellow at everything, especially in roughing it in the new countries to which all classes have emigrated from the beginning of the English colonies. It is because the descendants of the very best blood of Europe, and of England in particular, have dug and delved and sowed and reaped from Maine to Georgia for over two centuries, and still give dignity to labor in all parts of America by uniting it to refinement and intelligence, that we have the elevating tone of American thought and feeling. You cannot create a "gentle" man except out of a refined and gentle nature. Wealth, the tailor, the University, and "society" can only put on a transparent surface polish if there is not the hereditary elevating instinct which nothing can smother; and the snob's descendants invariably go back, sooner or later, to the class from which he sprung; when, for a certainty, their last state is worse than their first. It is, therefore an entire mistake to suppose the peasantry of any country have better teeth than the gentry, or are in any respect their superior. It is quite the other way. The English are divided into the upper, lower and middle classes, or, as Adam Smith puts it, into those who live by rents on land, those who live by labor, and those who live by profits. The upper class is distinguished by simplicity of manners and of personal living, cleanliness, high integrity, and great frugality. With plenty of fresh air and exercise, and a simple diet, they are very strong and have excellent teeth. The people of the lower class are uncleanly, eat their food miserably cooked, are passionately fond of dainties, are imprudent in all their doings, and so improvident that, as a rule, they can not lay out their earnings so as to make them spread over a single week, but want food before the new wages come in. The agricultural laborers get plenty of fresh air, and from dire necessity have a simple diet, and in consequence somewhat overcome the evil results of their instincts, and have fairly good teeth. But the artisans among the lower orders have not these compensating benefits, and the effects of their faults are intensified by living in large towns, working in impure air, and above all, by having good wages to spend in indulging their appetites. The consequence is that they have bad teeth. The middle classes is a thoroughly mongrel race, made up from all ranks and classes. It consists of the merchant, farmer, professional man, tradesman, etc. This class is as mixed in England as in the colonies. The uncertainties of all profit causes immense changes in each generation. The great merchant may be the son of a farm laborer and his clerk may be grandson of a lord. It is, therefore, but natural that we find in the middle class all sorts and conditions of teeth, from the very best to the very poorest. It will be a mistake to suppose that because the teeth are bad we can say the patient is from a low family, for I have known poor teeth to go with the longest pedigree; and among the artisans I have seen splendid teeth. It is only the general average I have been giving. The Welch peasantry have the poorest teeth I have ever met in Europe. They are pearly and pretty in youth, but soon decay. The lowest class of Germans have large teeth, as do most inferior people, which serve them fairly well so long as they live out of doors and eat wholesome bread. But they are deficient in vitality, have but little stamina, and if attacked by decay crumble away rapidly. The upper class of Germans have good teeth, but not so vital as the English or the Americans. The French of Gothic origin (see Magitot) have teeth much like the Germans; but those of Keltic origin have vastly better teeth. There cannot be a doubt that among the pre-historic people of Europe the Kelts had the best teeth. The Irish are a curiously mixed people. The peasantry, who are the descendants of the aborigines, have coarse, large and not good teeth. All around the coast of Ireland there settled in ancient times, the Northmen, and their descendants have good and strong teeth. Then a very much larger proportion of the Irish are English in origin than is admitted, and their teeth are much like the English. The North of Ireland is almost wholly of Scotch descent, and here we find good, strong, and vital teeth. There is even a more marked difference in Scotland than in England between the different classes. The Scotch peasant has fairly good teeth, and better than his English neighbor. Among the higher classes of Scotland we find the teeth fine in form, compact in structure, and highly organized. In all probability the aristocracy of Scotland is the finest race in Europe. The Danes, Swedes and Norwegians, being a superior race, have fine teeth. The Spanish teeth decay early, and the Portugese still sooner. The Greeks (I mean the real Greeks) have good teeth, as a rule. They compare well with the English, and so do the Turks and Arabs. What little experience I have had with the woolly-haired Africans shows they have very poor and dark yellow teeth, not white, as is so persistently asserted, with unusually large roots. Now, how do these teeth compare with the Americans? No man who will take the trouble to make careful observations will, I think, come to any other conclusion than that, on the whole, the teeth of the Americans, for strength, fineness of structure, and vitality, are decidedly superior to those of any other people. And conservative dentistry flourishes in that country, not because the teeth are unusually bad and need more than usual attention, but from the very opposite reason. It is because the teeth of the Americans present a very much larger proportion, than that of other people, of those which experience shows the dentist can save by proper operations. The great drawback to conservative dentistry in other parts of the world is found in the fact that the teeth are, except among the better classes, relatively poorer, and can not be saved by the same skill as can the American's. There are more dentists in America because of the general superiority of the teeth, the natural desire to save them, and the comparative ease with which this is accomplished. And dentistry will remain, in most other parts of the world, because of the nature of the teeth of the mass of the population, to a very great extent very much what it always has been, the means for supplying the inevitable false ones.—*Archives of Dentistry.*

---

ARTICLE III.

